# DEVELOPING TRAUMA-INFORMED APPROACHES IN PUBLIC HOUSING COMMUNITIES FOR OLDER PERSONS

**DOI:** 10.1093/geroni/igz038.2959

**Published:** 2019-11-08

**Authors:** Sharon Bowland, Becky Knight

**Affiliations:** University of North Texas, Denton, Texas, United States

## Abstract

In a study using a grounded theory approach with trauma survivors living in a mixed-age public housing community in Louisville, KY, twenty-five older women (50+) identified their main concern as housing safety. The study underlined the importance of community-level effects on health and well-being. Additionally, almost all participants identified that they had multiple experiences of interpersonal trauma across the life course. There may be increased negative health effects for older women living in public housing based on a history of interpersonal trauma. First, we will examine how women living in the community coped with their safety concerns. Second, we will explore policy implications of older trauma survivors living in communities that may exacerbate their trauma histories due to an ongoing lack of safety. Finally, we will consider how implementing trauma-informed approaches in public housing can potentially lead to improved health and well-being for those living with complex trauma.

